# Final follow-up of the Multicentre Aneurysm Screening Study (MASS) randomized trial of abdominal aortic aneurysm screening

**DOI:** 10.1002/bjs.8897

**Published:** 2012-12-03

**Authors:** S G Thompson, H A Ashton, L Gao, M J Buxton, R A P Scott, Multicentre Aneurysm Screening Study (MASS) Group

**Affiliations:** 1 Department of Public Health and Primary Care, University of CambridgeUK; 2 Medical Research Council Biostatistics UnitCambridge, UK; 3 Sussex Community NHS Trust, St Richard's HospitalChichester, UK; 4 Western Sussex Hospitals NHS Trust, St Richard's HospitalChichester, UK; 5 Health Economics Research Group, Brunel UniversityUxbridge, UK

## Abstract

**Background:**

The long-term effects of abdominal aortic aneurysm (AAA) screening were investigated in extended follow-up from the UK Multicentre Aneurysm Screening Study (MASS) randomized trial.

**Methods:**

A population-based sample of men aged 65–74 years were randomized individually to invitation to ultrasound screening (invited group) or to a control group not offered screening. Patients with an AAA (3·0 cm or larger) detected at screening underwent surveillance and were offered surgery after predefined criteria had been met. Cause-specific mortality data were analysed using Cox regression.

**Results:**

Some 67 770 men were enrolled in the study. Over 13 years, there were 224 AAA-related deaths in the invited group and 381 in the control group, a 42 (95 per cent confidence interval 31 to 51) per cent reduction. There was no evidence of effect on other causes of death, but there was an overall reduction in all-cause mortality of 3 (1 to 5) per cent. The degree of benefit seen in earlier years of follow-up was slightly diminished by the occurrence of AAA ruptures in those with an aorta originally screened normal. About half of these ruptures had a baseline aortic diameter in the range 2·5–2·9 cm. It was estimated that 216 men need to be invited to screening to save one death over the next 13 years.

**Conclusion:**

Screening resulted in a reduction in all-cause mortality, and the benefit in AAA-related mortality continued to accumulate throughout follow-up. Registration number: ISRCTN37381646 (http://www.controlled-trials.com).

## Introduction

National screening programmes for abdominal aortic aneurysm (AAA) have recently been initiated for men in England and Scotland^1^, ^2^, Sweden^3^, and the USA as part of Medicare^4^. The UK Multicentre Aneurysm Screening Study (MASS)^5–7^ has provided the majority of the worldwide randomized evidence, in terms of both number of participants and person-years of follow-up, for the mortality benefit following ultrasound screening for AAA^8^, ^9^. The English screening programme for men aged 65 years is based closely on the protocol and procedures in the MASS trial. However, there remain some uncertainties relating to AAA screening, including its long-term mortality benefit, and whether rescreening of those with a previously normal scan is warranted. It might be expected that the mortality benefit seen in the early years after one-off screening would decrease over time. The MASS trial, started in 1997, runs more than 10 years ahead of the English national screening programme, and is uniquely positioned to address these uncertainties and inform the development of national programmes.

Results from MASS were last published after 10 years of follow-up^7^; some increase in ruptured AAA among those screened normal was noted, but this had not impacted on the overall proportionate reduction in AAA-related mortality. The only existing randomized trial evidence after 10 years comes from much smaller trials; the 14-year follow-up of the Danish Viborg trial did not report on ruptured AAA among those screened normal^10^, whereas the 15-year follow-up of the UK Chichester trial suggested a possibly substantial increase in ruptured AAA during later follow-up^11^. Such an increase would reduce the long-term benefit from a single initial scan. To provide more reliable evidence, new information is now available from the MASS trial.

## Methods

The design of the MASS trial has been described in detail elsewhere^5^. In brief, a population-based sample of men aged 65–74 years were recruited in 1997–1999 from four centres in the UK, and randomized to receive an invitation to screening (invited group) or not (control group). Randomization was conducted centrally using computer-generated pseudo-random numbers, stratified by centre and general practice. The anterior–posterior and transverse internal diameters of the abdominal aorta were measured ultrasonographically, and the higher value was recorded as the aortic diameter. Men with an aortic diameter of 3 cm or greater were diagnosed with an AAA. Aortas with a smaller diameter were classified as normal (recorded simply as less than 3·0 cm) and these men were not recalled for further imaging. Within the group of detected AAAs, surveillance involved repeat imaging annually for aneurysms with an aortic diameter of 3·0–4·4 cm and every 3 months for those with a diameter of 4·5–5·4 cm. Referral to a hospital vascular department for consideration for elective surgery was made when the aortic diameter reached 5·5 cm, aortic expansion was 1·0 cm or more in 1 year, or symptoms attributable to the aneurysm were reported.

Additional data were collected from local hospital records relating to ultrasound scans and AAA surgery. Deaths up to 31 March 2011 were notified by the UK Office for National Statistics following matching on the unique National Health Service (NHS) number for each individual. Follow-up ranged from 11·9 to 14·2 (mean 13·1) years. The primary outcome of interest, AAA-related mortality, was defined as all deaths within 30 days of any AAA operation (elective or emergency) plus all deaths with International Classification of Diseases (ICD) 9 codes 441.3–441.6. Where possible, the baseline scans were retrieved for men screened normal who subsequently had an AAA rupture, and the aortic diameter was remeasured (to give an exact result in millimetres) by a single experienced radiologist.

Extended follow-up in MASS was approved by Southampton and South West Hampshire Ethics Committee (A), March 2007.

### Analysis

AAA-related mortality (censoring other causes of death) and all-cause mortality were compared between the two randomized groups using unadjusted Cox regression. An unbiased randomization-based estimate of the benefit of attending initial screening was also obtained^12^. This estimate was calculated by subtracting from the controls a group equivalent to the non-attending group among those invited, leaving a control group comparable with those attending in the invited group. The effects of age on contraindication to, or refusal of, elective surgery and on 30-day mortality after elective surgery were estimated by logistic regression, taking into account calendar year and, for 30-day mortality, group (control, invited attendee, invited non-attendee) and type of AAA surgery (open or endovascular aneurysm repair).

## Results

Of a total of 70 495 men aged 64–75 years, 67 770 were randomized (details in Supplementary *Fig. S1*). The randomized groups were well balanced in terms of age, geographical area and socioeconomic status^5^, ^13^. Among the 33 883 men invited to screening, principally in a primary care setting, 27 204 (80·3 per cent) attended. Of the 1334 men (4·9 per cent prevalence) with an AAA detected at the initial scan, 942 (70·6 per cent) had complete clinical follow-up to 13 years according the protocol; about half of the losses occurred during the first 3 years of the trial. Follow-up for mortality was almost complete (97·1 per cent); the remainder (mainly those who had moved abroad) were censored at the time last known to be alive. The mean age of the men in MASS at the end of follow-up was 83 years.

Among the 1334 patients with an AAA detected at screening, 820 had died by the end of follow-up, of whom 477 (58·2 per cent) neither had a ruptured aneurysm nor had undergone AAA repair. Of the 514 who were still alive at the end of follow-up, 216 (42·0 per cent) had not undergone AAA surgery. There were about twice as many elective AAA operations in the invited group as in the control group (600 *versus* 277), but only half the emergency operations (80 *versus* 166) (*Table* *1*). The overall 30-day mortality rate after elective surgery was 4·2 per cent (37 of 877); it increased with age at operation (odds ratio per 5 years 1·6, 95 per cent confidence interval (c.i.) 0·9 to 2·9), but did not differ between the invited and control groups (*P* = 0·403).

**Table 1:** Operations for abdominal aortic aneurysm over 13 years and subsequent 30-day mortality in the Multicentre Aneurysm Screening Study

	Control group		Invited group	
	Operations	Deaths	Operations	Deaths
Elective surgery	277	14 (5·1)	600	23 (3·8)
Through screening programme	0		536	17
Not through screening programme*	277	14	64	6
Emergency surgery	166	57 (34·3)	80	27 (34)
For ruptured AAA	144	54	59	23
For symptomatic AAA	22	3	21	4
Other†	2	0	14	1

Values in parentheses are percentages.

* In non-attendees at initial scan or in those not undergoing regular surveillance.

† Operations primarily for iliac aneurysm, with abdominal aortic aneurysm (AAA) surgery done at the same time.

Only 12·8 per cent of the elective operations were by endovascular aneurysm repair: the 30-day mortality rate was 1·8 per cent (2 of 112) after endovascular and 4·6 per cent (35 of 765) after open repair. The overall turn-down rate (contraindications and refusals) for elective surgery was 14·6 per cent (115 of 787); it increased with age at consultation (odds ratio per 5 years 1·6, 95 per cent c.i. 1·1 to 2·3). The 30-day mortality rate after emergency surgery was 34·1 per cent (84 of 246), with similar rates in each randomized group (*Table* *1*).

**Figure 1:**
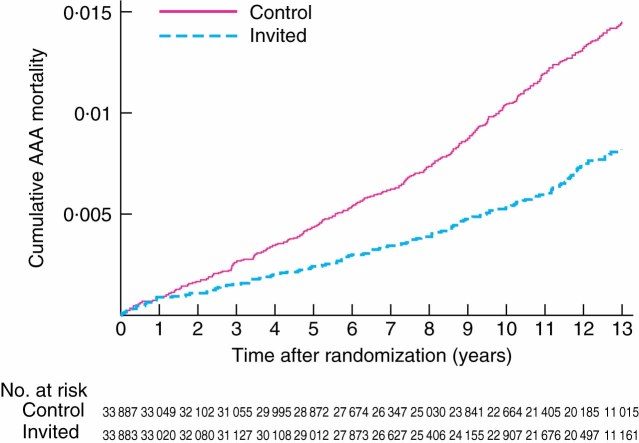
Abdominal aortic aneurysm (AAA)-related mortality over 13 years in the Multicentre Aneurysm Screening Study

There were 224 AAA-related deaths (absolute risk 0·66 per cent) in the invited group compared with 381 (1·12 per cent) in the control group, a relative risk reduction of 42 per cent (hazard ratio 0·58, 95 per cent c.i. 0·49 to 0·69) (*Table* *2*). There was no evidence that the relative risk reduction varied by baseline age or centre (tests for interaction *P* = 0·586 and *P* = 0·531 respectively). The number of men needed to be invited to screening to save one death over 13 years was estimated as 216. The AAA mortality curves in the two groups continued to diverge throughout follow-up (*Fig.* *1*). However, there was a slight reduction in the benefit seen after 10 years; from randomization to 10 years the risk reduction was 48 per cent, whereas during years 10–13 it was 20 per cent.

**Table 2:** Abdominal aortic aneurysm-related mortality, rupture and all-cause mortality over 13 years in the Multicentre Aneurysm Screening Study

	Control group (*n* = 33 887)	Invited group (*n* = 33 883)
Person-years of follow-up	350 800	353 100
AAA-related deaths		
Within 30 days of	14	24
elective operation‡		
From ruptured AAA§	327	170
From AA rupture,	40	30
unspecified site¶		
Total*	381 (1·09)	224 (0·63)
Hazard ratio†	1·00 (reference)	0·58 (0·49, 0·69)
Ruptured AAA		
Non-fatal#	95	49
Total* **	476 (1·36)	273 (0·77)
Hazard ratio†	1·00 (reference)	0·57 (0·49, 0·66)
Non-AAA causes of death		
Ischaemic heart disease	3049	2935
Stroke	926	918
Other cardiovascular	1050	1076
Cancer	4768	4755
Other††	3960	3950
All-cause deaths*	14 134 (40·3)	13 858 (39·2)
Hazard ratio†	1·00 (reference)	0·97 (0·95 to 0·99)

Values in parentheses are ^*^rate per 1000 person-years and

† 95 per cent confidence intervals.

‡ Includes those with International Classification of Diseases (ICD) 9 codes 441·3–441·6 who died within 30 days of elective abdominal aortic aneurysm (AAA) surgery;

§ ICD-9 codes 441·3 (ruptured AAA) and 441·4 (AAA without mention of rupture), and all deaths occurring within 30 days of emergency AAA surgery;

¶ aortic aneurysm deaths with ICD-9 codes 441·5 (ruptured aortic aneurysm at unspecified site) and 441·6 (aortic aneurysm at unspecified site without mention of rupture).

# Excludes nine men with non-fatal ruptured AAA in control group and one in invited group who later died from ruptured AAA.

** AAA-related deaths plus non-fatal ruptured AAA.

†† Includes 75 deaths of unknown cause.

Over the whole 13-year interval, non-fatal AAA ruptures were also approximately halved in the invited group (*Table* *2*). The numbers of non-AAA-related deaths were similar between randomized groups (*Table* *2*); the reduction in ischaemic heart disease deaths in the invited group was not statistically significant (*P* = 0·080). Overall, there was a 3 per cent reduction in all-cause mortality in the invited group (hazard ratio 0·97, 0·95 to 0·99).

The unbiased estimate of the 13-year reduction in AAA-related mortality among men who attended screening was 52 per cent (hazard ratio 0·48, 0·40 to 0·59). This estimate is relevant when providing information to individuals about the benefit of screening, or when considering the benefit from a screening programme that achieves an attendance rate different from the 80 per cent achieved in MASS.

In the invited group, 24 men died within 30 days of elective surgery and another seven after more than 30 days (2 in-hospital deaths and 5 later AAA-related deaths). In total, 273 men died after elective surgery or had a ruptured AAA despite being invited for screening (*Table* *3*). Many of these were among those who did not attend screening (86 men), those who did not keep a follow-up appointment for routine surveillance or vascular assessment (27), those who refused surgery (6) and those considered unfit for surgery (17). Some AAA ruptures occurred between recall scans (17), during surveillance reassessment (4), pending a decision regarding surgery (17) and while awaiting surgery (10).

**Table 3:** Timing of abdominal aortic aneurysm ruptures and deaths in 33 883 men invited to screening

	Non-fatal AAA ruptures (*n* = 49)	AAA-related deaths (*n* = 224)	Total (*n* = 273)
AAA not identified by screening programme			
Between randomization and scan	0	3	3
After non-attendance at screening (*n* = 6679)*	11	75	86
After non-visualized first scan (*n* = 329)	1	1	2
After normal first scan (*n* = 25 541)†	12	49	61
AAA identified by screening programme			
AAA < 5·5 cm‡ (*n* = 727)			
Between recall scans	7	10	17
After non-attendance for surveillance	1	24	25
AAA ≥ 5·5 cm‡ (*n* = 607)			
After non-attendance for vascular assessment	0	2	2
After refusal of elective surgery	0	6	6
After being declared unfit for elective surgery	0	17	17
Pending decision regarding elective surgery	9	8	17
While awaiting elective surgery	7	3	10
After return to surveillance for reassessment§	1	3	4
After elective surgery			
⩽ 30 days after elective surgery	0	17	17
> 30 days after elective surgery	0	6	6

* Includes six deaths after elective operations (5 within 30 days) following incidental detection of abdominal aortic aneurysm (AAA).

† Includes two deaths within 30 days of elective surgery following incidental detection of AAA.

‡ Aneurysm size based on the maximum observed from all scans.

§ Aneurysm of at least 5·5 cm not confirmed at outpatient visit.

**Figure 2:**
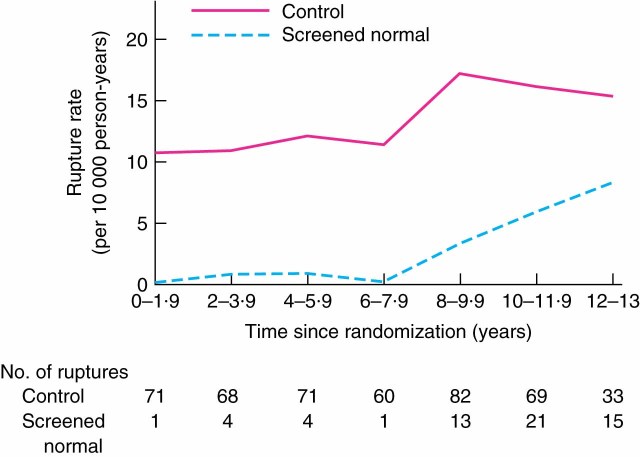
Rate of ruptured abdominal aortic aneurysm in men who originally screened normal and in the control group over time

**Figure 3:**
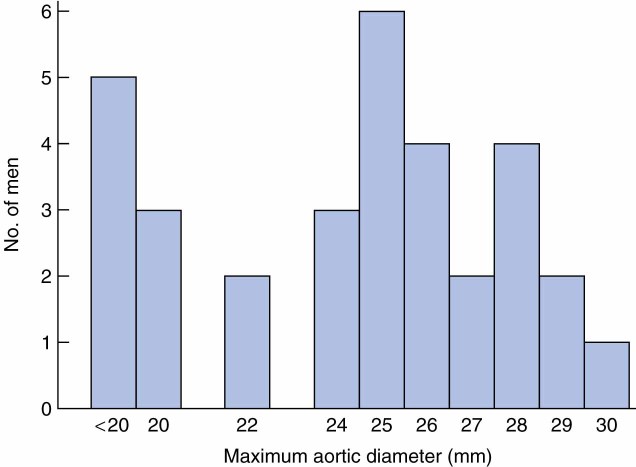
Distribution of baseline aortic diameter among 32 men with ruptured abdominal aortic aneurysm who originally screened normal

A total of 59 ruptured AAAs occurred after a normal first scan, of which 47 (80 per cent) were fatal. These 59 men had a mean age of 68·7 years at screening, the same as that for all the other men who had a normal first scan. There was a marked increase in the rate of these ruptured aneurysms after 8 years of follow-up. By years 12–13 the rate had increased to 8·3 per 10 000 person-years, but remained substantially lower than the overall rate of AAA rupture in the control group (*Fig.* *2*). Time since initial scan, rather than age, was the determinant of this increased risk of rupture. It was possible to retrieve 32 of the baseline scans for the 59 men with such a rupture (baseline scans were not available in one centre). Among the remeasured baseline aortic diameters, about half (18 of 32) were in the range 2·5–2·9 cm (*Fig.* *3*).

## Discussion

The benefit of being invited to screening continued to accumulate throughout the 13-year follow-up in the MASS trial. The risk of AAA-related mortality and AAA rupture was almost halved, and there was a small but convincing reduction in all-cause mortality. The number needed to be invited to screening to save one AAA-related death over 13 years was estimated as 216, better than, for example, corresponding figures quoted for breast cancer screening (about 400 or more)^14^. Put another way, each 10 000 men screened (requiring 12 500 to be invited, assuming an 80 per cent attendance rate) saved 75 ruptured AAAs (58 fatal), leading to an extra 119 elective AAA procedures, with an associated five postoperative deaths, over 13 years. The overall balance of benefit to harm in terms of mortality was in favour of screening.

The mortality benefit was somewhat less in years 10–13 after screening than before year 10. This was at least partly due to ruptured AAA in men originally screened normal, with a steady increase in these ruptures from year 8 onwards; the large majority of these were fatal. Although the rate was small in absolute terms (less than 1 per 1000 per year even at years 12–13), it was enough to diminish the benefit of AAA screening during this late follow-up. In addition, there was a cumulative effect of incidental AAA detection in the control group, as apparent through the elective operations taking place, and a greater operative mortality and contraindication/refusal rate for elective surgery as age increased. All these aspects may have contributed to the reduced mortality benefit later in follow-up.

The only apparent determinants of the increased risk of AAA rupture in those screened normal were the aortic diameter at baseline and the time since screening. About half of these ruptures were in men with an initial aortic diameter of 2·5–2·9 cm. Based on other population screening programmes in the UK, the proportion of men aged 65 years with an internal aortic diameter in the range 2·5–2·9 cm is less than 3 per cent: it reduced from about 3 per cent to 1 per cent over 1991–2009 in Gloucestershire^15^, was 2·6 per cent in Chichester and Worthing in 2004–2007^16^, and 1·6 per cent in the English national screening programme in 2009–2011^1^. In programmes measuring external aortic diameters, the proportion would be expected to be somewhat higher. Although the men in MASS had a mean age of 69 years at screening^5^ rather than 65 years, the increase in AAA prevalence with age is modest^13^. Thus there appears to be a large difference in the proportions of men with 2·5–2·9-cm aortic diameters: among the men in MASS with an initially normal scan whose aneurysm later ruptured it was about half, whereas the background prevalence is less than 3 per cent.

To reduce late ruptures in future, two policies are possible. The first would be to rescreen all men with a normal aortic diameter, for example after 10 years, but this would be costly. The second would be to include those with an aortic diameter in the range 2·5–2·9 cm in the surveillance programme. It would be sufficient to recall these men only after, for example, 5 years in the first instance, because the chance of developing an AAA larger than 5·5 cm or having an AAA rupture before that time is very small^17–21^. Hence the additional cost to the surveillance programme would be modest.

Being based on a population-based sample of UK men, the results from MASS should in principle correspond with the expected benefit that will derive from the NHS AAA Screening Programme (NAAASP). However, the prevalence of AAA (diameter at least 3·0 cm) is about 1·7 per cent in NAAASP^1^, compared with 4·9 per cent in MASS, and the number of ruptured AAAs is currently falling in the UK and elsewhere^22–25^. The reasons for the lower prevalence are not fully understood, but may partly be because NAAASP is targeting a younger age (men aged 65 years, rather 65–74 years as in MASS), in some NAAASP centres the ethnic mix is different, and the uptake is lower than in MASS. In addition, there has been a reduction in smoking prevalence and a general reduction in cardiovascular and atherosclerotic diseases over the intervening 15 years, together with an increased use of some drugs (especially statins) and of elective surgery for those aged over 75 years^26^, ^27^. Therefore, although it would still be expected that about half of the AAA-related deaths in men aged 65 years and over in the UK should eventually be prevented, the absolute benefit will not be as large as in MASS.

Based on MASS data, AAA screening is a cost-effective policy in NHS terms. The cost per life-year gained was estimated as £7600 at 10 years^7^, and using health economic modelling as £2300 over the lifetime of men aged 65 years^28^. These figures are both below the guideline threshold figure of around £20 000 per life-year gained for the acceptance of medical technologies by the National Institute for Health and Clinical Excellence in the UK^29^. Although a formal analysis of the effects of the uptake rate, observed prevalence and aneurysm size distribution, and current costs of the screening and operative procedures is needed, it is expected that the NHS AAA screening programme will still be clearly cost-effective. This is because the main cost drivers are the elective and emergency AAA operations^30^, and these (together with preoperative surveillance costs for small AAAs) will be reduced roughly in proportion to the number of AAAs detected.

Some potential limitations of the present analyses of the MASS trial should be mentioned. The inclusion of deaths from aortic aneurysm at an unspecified site may have provided a conservative estimate of the benefit of screening, because the use of ICD-9 codes 441.5 and 441.6 may have resulted in the inclusion of some non-abdominal aortic (such as thoracic) aneurysms. Investigation of the accuracy of cause of death coding on the death certificates in the first 4 years of follow-up was carried out by an independent mortality working party blinded to group allocation, and showed that inaccuracies in coding had a minimal impact on study outcomes^5^. The quality-of-life data collected in the trial around the time of screening showed no clear adverse (or beneficial) effects of screening, nor any long-term effects after surgery^5^, ^31^.

Although the loss to mortality follow-up was small as patients were tracked through their NHS number, full follow-up for surgical AAA operations was more difficult to achieve. Surgical follow-up was through data review of AAA operations performed at the local hospitals in each screening area. Operations among patients who had moved away or had surgery at other hospitals were therefore missed, and subsequent deaths within 30 days of the operation would not necessarily have been recorded as AAA-related on the death certificate. The extent of this problem was estimated in one MASS centre, by calculating from information on death certificates the number of deaths in hospital that occurred outside the area. This gave a rate of 419 of 6116 (6·9 per cent); being a small proportion, it indicates that only a few people in this age group moved out of the area and were lost to surgical follow-up. Consequently, these potentially missed data should have had only a minor impact on the trial results.

Long-term follow-up of the MASS trial has shown that it is possible to achieve almost a halving of the AAA-related mortality rate by screening men aged 65–74 years. Rescreening of all those originally screened normal is not justified, but consideration should be given in future to offering a further scan after about 5 years for the small proportion of men with an aortic diameter in the range 2·5–2·9 cm.

## Notes

Fig. S1 CONSORT diagram for the MASS trial (Word document)

Please note: John Wiley & Sons Ltd is not responsible for the functionality of any supporting materials supplied by the authors. Any queries (other than missing material) should be directed to the corresponding author for the article.
