# Supplementary material for: Final follow-up of the Multicentre Aneurysm Screening Study (MASS) randomized trial of abdominal aortic aneurysm screening
Source: Br J Surg. 2012 Dec 3;99(12):1649–56. doi: 10.1002/bjs.8897 (PMC3569614; doi:10.1002/bjs.8897)
Supplement: Fig. S1 — CONSORT diagram for the MASS trial (Word document) [file bjs0099-1649-sd1.doc]

**BJS8897**

**Final follow-up of the Multicentre Aneurysm Screening Study (MASS) randomized trial of abdominal aortic aneurysm screening**

S. G. Thompson, H. A. Ashton, L. Gao, M. J. Buxton and R. A. P. Scott on behalf of the Multicentre Aneurysm Screening Study (MASS) Group


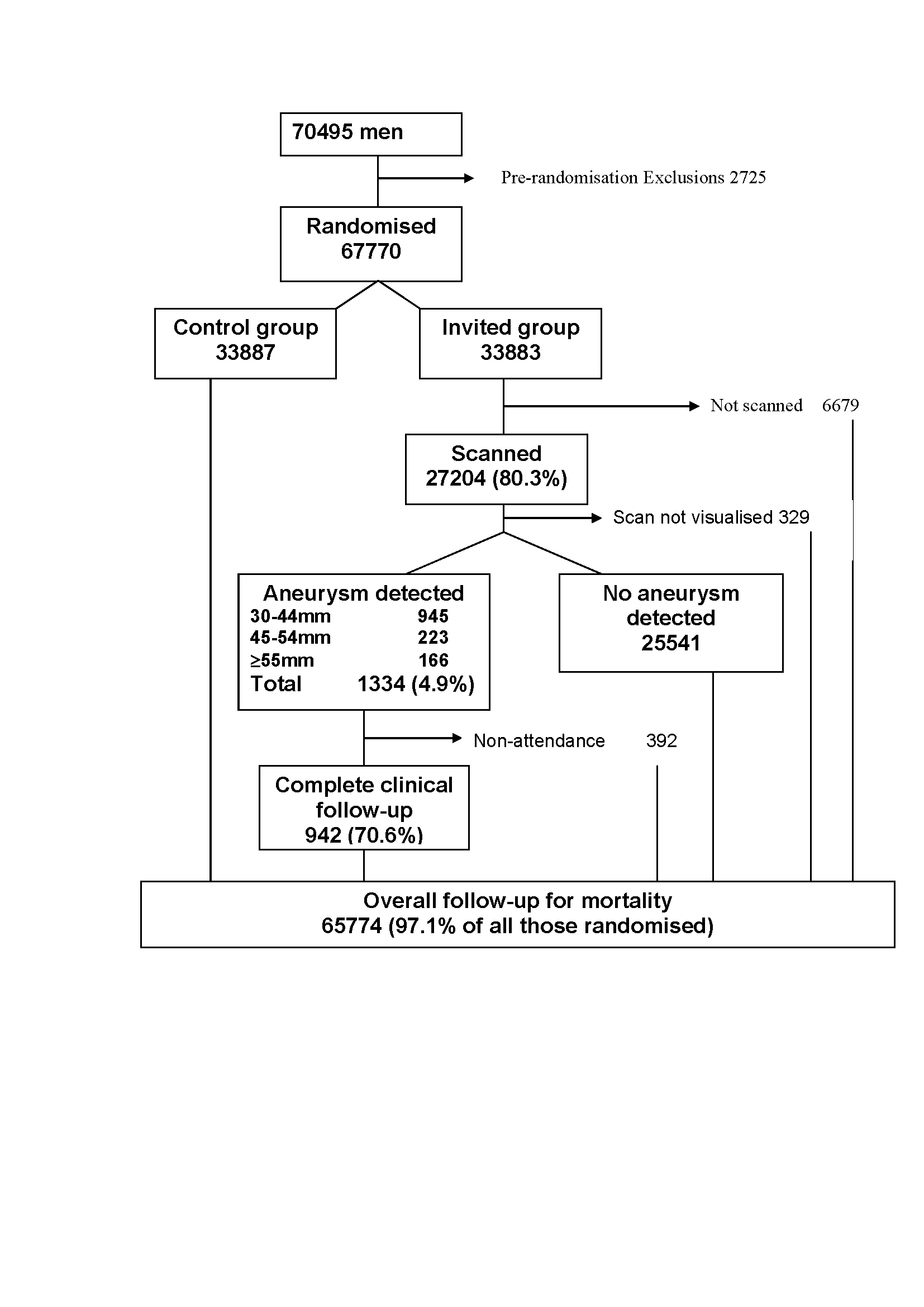


**Fig. S1** CONSORT diagram for the MASS trial. Percentages are based on number in previous box, except for overall mortality follow-up
